# Effectiveness of the Rigo Chêneau versus Boston-style orthoses for adolescent idiopathic scoliosis: a retrospective study

**DOI:** 10.1186/s13013-017-0117-z

**Published:** 2017-03-20

**Authors:** Miriam K. Minsk, Kristen D. Venuti, Gail L. Daumit, Paul D. Sponseller

**Affiliations:** 1 Department of Orthopaedic Surgery, The Johns Hopkins University, Baltimore, MD USA; 2 Division of General Internal Medicine, The Johns Hopkins University School of Medicine, Baltimore, MD USA; 3 Welch Center for Prevention, Epidemiology, and Clinical Research, The Johns Hopkins University, Baltimore, MD USA; 4 Department of Epidemiology, The Johns Hopkins Bloomberg School of Public Health, Baltimore, MD USA; 5 Department of Health Policy and Management, The Johns Hopkins Bloomberg School of Public Health, Baltimore, MD USA; 6 Bloomberg Children’s Center, 1800 Orleans Street, 7359A, Baltimore, MD 21287 USA

**Keywords:** Adolescent, Bracing, Major curve, Orthosis, Outcomes, Scoliosis

## Abstract

**Background:**

Bracing can effectively treat adolescent idiopathic scoliosis (AIS), but patient outcomes have not been compared by brace type. We compared outcomes of AIS patients treated with Rigo Chêneau orthoses (RCOs) or custom-molded Boston-style thoracolumbosacral orthoses (TLSOs).

**Methods:**

We retrospectively reviewed patient records from one scoliosis center from 1999 through 2014. Patients were studied from initial treatment until skeletal maturity or surgery. Inclusion criteria were a diagnosis of AIS, initial major curve between 25° and 40°, use of an RCO or TLSO, and no previous scoliosis treatment.

**Results:**

The study included 108 patients (93 girls) with a mean (±standard deviation) age at brace initiation of 12.5 ± 1.3 years. Thirteen patients wore an RCO, and 95 wore a TLSO. Mean pre-bracing major curves were 32.7° ± 4.8° in the RCO group and 31.4° ± 4.4° in the TLSO group (*p* = 0.387). Mean brace wear time was similar between groups. Mean differences in major curve from baseline to follow-up were −0.4° ± 9.9° in the RCO group and 6.9° ± 12.1° in the TLSO group (*p* = 0.028). Percent changes in major curve from baseline to follow-up were 0.0% ± 30.5% for the RCO group and 21.3% ± 38.8% for the TLSO group (*p* = 0.030). No RCO patients and 34% of TLSO patients progressed to spinal surgery (*p* = 0.019). At follow-up, major curves improved by 6° or more in 31% of the RCO group and 13% of the TLSO group (*p* = 0.100).

**Conclusions:**

Patients treated with RCOs compared with Boston-style TLSOs had similar baseline characteristics and brace wear time yet significantly lower rates of spinal surgery. Patients with RCOs also had lower mean and percent major curve progression versus those with TLSOs.

## Background

Adolescent idiopathic scoliosis (AIS) affects 2 to 3% of adolescents between the ages of 10 and 18 years [1, 2]. Brace treatment is commonly offered when the spinal curve has reached 25° [3]. Since the Bracing in Adolescent Idiopathic Scoliosis Trial study in 2013 [4], bracing has been increasingly recognized as an effective nonsurgical means of scoliosis treatment. However, the comparative effectiveness of most types of braces for AIS has not been definitively established [5].

A rigid thoracolumbosacral orthosis (TLSO) is a brace worn to minimize progression of AIS. There are various TLSO designs (e.g., Boston, Milwaukee, Wilmington) [6]. Rigo Chêneau orthoses (RCOs) were developed approximately two decades ago, with the intent to combine biomechanical forces in three dimensions, including curve derotation. They use an open pelvis design with anterior opening. However, studies of the RCO are limited, and we know little about its effectiveness, particularly in relation to other braces [5, 7, 8].

In the current study, we reviewed records of patients treated at one large academic medical center’s pediatric orthopedic scoliosis practice who were prescribed full-time bracing for AIS. Our objective was to determine if brace type, specifically the RCO compared with a Boston-style TLSO, affected outcomes. Our hypothesis was that different brace designs would lead to different patient outcomes.

## Methods

### Study population

We retrospectively reviewed medical records of patients treated at an academic scoliosis center from 1999 through 2014. The study population consisted of adolescents aged 10 years or older at presentation who met the following criteria: (1) diagnosis of AIS; (2) Risser stage between 0 and 2; (3) major curve between 25° and 40°; (3) no previous treatment for scoliosis; (4) if female, premenarchal or less than 1 year postmenarchal; (5) prescribed full-time brace treatment; and (6) follow-up until skeletal maturity or surgery.

### Measurements

Outcome variables followed the recommendations of the Scoliosis Research Society (SRS) Committee on Bracing and Nonoperative Management and the Society on Scoliosis Orthopaedic and Rehabilitation Treatment (SOSORT) and incorporated other relevant clinical outcomes [9, 10]. Outcomes included the following: major curve exceeding 30° and major curve exceeding 50°, difference in major curve from baseline to follow-up, percent change in major curve, progression to spinal surgery, progression of curve to 45° or more after bracing, progression to spinal surgery or curve of at least 45° after bracing, major curve progression of 6° or more, major curve improvement of 6° or more, and major curve unchanged (within 5°). For the outcomes that included progression to curvature of 45° or more, we measured the patients whose major curve progressed to at least 45°.

Our primary independent variable was the type of brace. We compared an RCO with a custom Boston-style TLSO. Patients self-selected their orthotists and brace type. Follow-up orthopedist recommendations were the same for all patients: in-brace radiography and clinic visit 4 weeks after treatment initiation, then out-of-brace radiography and clinic visits every 4 months before menarche and every 6 months after menarche. We abstracted information on age, sex, race, curve location, pre-bracing initial major curve magnitude, pre-bracing Risser stage, initial in-brace major curve, time in brace, and mean patient-reported number of hours the brace was worn in Risser stages 0 and 1 and overall. We recorded information for the total course of treatment for each patient and calculated the mean brace wear time for the course of treatment.

We performed univariate and bivariate descriptive analyses, including Student *t* tests, Fisher exact tests, and *χ* ^2^ tests, comparing baseline characteristics and outcomes. A two-sided alpha with *p* < 0.05 was considered statistically significant.

## Results

### Baseline characteristics

Of the 108 patients (93 girls) who met the inclusion criteria, the mean age at treatment initiation was 12.5 ± 1.3 years (Table 1). Ninety-five patients were treated with a TLSO, and 13 patients were treated with an RCO. Of the study population, 72% were Caucasian and 15% were African American. Major curves were mainly thoracic (47%), lumbar (22%), or thoracic and thoracolumbar (18%). The mean pre-brace major curves were 31.6° ± 4.4° overall, 32.7° ± 4.8° in the RCO group, and 31.4° ± 4.4° in the TLSO group, corresponding to 52% of patients having an initial pre-brace major curve of more than 30°. Sixty-three percent of patients began bracing at Risser stage 0, 22% at Risser stage 1, and 15% at Risser stage 2. Demographic and clinical characteristics at baseline were similar for patients in both groups.

**Table 1:** Demographic and clinical characteristics at baseline for 108 patients with adolescent idiopathic scoliosis

Characteristics	Patients						
	All (*n* = 108)		RCO group (*n* = 13)		Boston-style TLSO group (*n* = 95)		*P*
	Mean (SD)	*n* (%)	Mean (SD)	*n* (%)	Mean (SD)	*n* (%)	
Age (years)	12.5 (1.3)		12.5 (1.3)		12.5 (1.3)		0.762
Female sex		93 (86)		11 (85)		82 (86)	1.00
Race							0.282
Caucasian		78 (72)		10 (77)		68 (72)	
African American		16 (15)		0 (0)		16 (17)	
Hispanic		1 (1)		0 (0)		1 (1)	
Asian/Pacific Islander		2 (2)		0 (0)		2 (2)	
Other		11 (10)		3 (23)		8 (8)	
Major curve location							
Thoracic		51 (47)		5 (38)		46 (48)	0.500
Thoracolumbar		3 (3)		0 (0)		3 (3)	1.00
Lumbar		24 (22)		1 (7.7)		23 (24)	0.290
Double major		0 (0)		0 (0)		0 (0)	
Double thoracic		9 (8)		3 (23)		6 (6)	0.075
Thoracic and thoracolumbar		19 (18)		4 (31)		15 (16)	0.238
Triple		2 (2)		0 (0)		2 (2)	1.00
Major curve (°)	31.6 (4.4)		32.7 (4.8)		31.4 (4.4)		0.387
Major curve >30°		56 (52)		7 (54)		49 (51)	0.878
Risser stage							0.710
0		68 (63)		7 (54)		61 (64)	
1		24 (22)		4 (31)		20 (21)	
2		16 (15)		2 (15)		14 (15)	

*RCO* Rigo Chêneau orthosis, *TLSO* thoracolumbosacral orthosis, *SD* standard deviation

### Treatment and outcomes

We followed all patients until skeletal maturity or progression to surgery, whichever came first. Mean initial in-brace major curves were 22.6° ± 6.4° in the RCO group and 22.6° ± 7.2° in the TLSO group (*p* = 0.924) (Table 2). In-brace correction of major curve from baseline of at least 35% was achieved in 42% of the RCO group and 36% of the TLSO group (*p* = 0.943, data not shown). Patients in the RCO group wore the brace for a mean 17.0 ± 6.1 h per day, and patients in the TLSO group wore the brace for a mean 16.1 ± 5.2 h per day (*p* = 0.641).

**Table 2:** Bracing treatment and outcomes for 108 patients with adolescent idiopathic scoliosis

Parameter	Patients						*P*
	All (*n* = 108)		RCO group (*n* = 13)		Boston-style TLSO group (*n* = 95)		
	Mean (SD)	*n* (%)	Mean (SD)	*n* (%)	Mean (SD)	*n* (%)	
Initial in-brace major curve^a^ (°)	22.8 (7.2)		22.6 (6.4)		22.6 (7.2)		0.924
Percent initial in-brace major curve correction^a^	28.4 (20.1)		31.5 (15.2)		27.8 (20.1)		0.538
Time in brace (year)	2.4 (1.4)		2.8 (0.9)		2.4 (1.4)		0.193
Brace wear time per day (h)							
All patients^b^	16.2 (5.3)		17.0 (6.1)		16.1 (5.2)		0.641
Patients with Risser stage 0 or 1^c^	17.0 (5.8)		18.9 (5.8)		16.8 (5.8)		0.296
Final major curve (°)	37.6 (13.3)		32.3 (10.4)		38.3 (13.5)		0.077
Final major curve							
>30°		70 (65)		6 (46)		64 (67)	0.133
>50°		18 (17)		1 (8)		17 (18)	0.464
Change in major curve from baseline^d^ (°)	6.0 (12.1)		−0.4 (9.9)		6.9 (12.1)		0.028
Percent change in major curve from baseline	18.6 (38.9)		0.0 (30.5)		21.3 (38.8)		0.030
Progression to surgery		32 (30)		0 (0)		32 (34)	0.019
At skeletal maturity							
Major curve ≥45°		32 (30)		2 (15)		30 (32)	0.337
Progression to surgery or major curve ≥45°		38 (35)		2 (15)		36 (38)	0.133
Major curve change							
Progression ≥6°		52 (48)		5 (38)		47 (49)	0.556
Decrease ≥6°		16 (15)		4 (31)		12 (13)	0.100
Unchanged (±5°)		40 (37)		4 (31)		36 (38)	0.764

*SD* standard deviation, *RCO* Rigo Chêneau orthosis, *TLSO* thoracolumbosacral orthosis

^a^ *n* = 83 (RCO, *n* = 12; TLSO, *n* = 70)

^b^ *n* = 107 (RCO, *n* = 13; TLSO, *n* = 94)

^c^RCO, *n* = 10; TLSO, *n* = 71

^d^ *n* = 95 (RCO, *n* = 11; TLSO, *n* = 84)

**Fig. 1:**
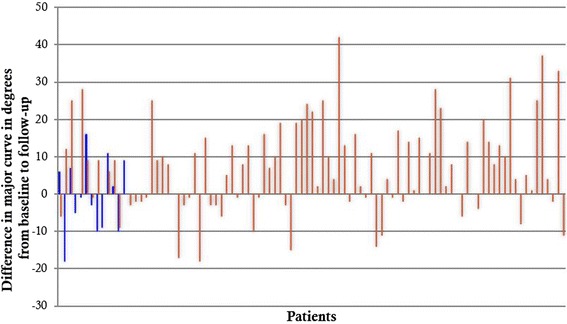
Difference in major curve after treatment with Rigo Chêneau orthoses (*blue lines*) compared with Boston-style thoracolumbosacral orthoses (*red lines*) in 108 patients with adolescent idiopathic scoliosis

After bracing was complete, the mean final measurements for major curves were 32.3° ± 10.4° (RCO group) and 38.3° ± 13.5° (TLSO group) (*p* = 0.077) (Table 2). Forty-six percent of RCO patients had a major curve at follow-up of greater than 30°, compared with 67% of TLSO patients (*p* = 0.133). The mean difference in major curves from baseline to follow-up was −0.4° ± 9.9° for the RCO group versus 6.9° ± 12.1° for the TLSO group (*p* = 0.028). Figure 1 shows each patient’s change in major curve magnitude from baseline to follow-up. The percent changes in major curves from baseline to follow-up were 0.0% ± 30.5% for the RCO group and 21.3% ± 38.8% for the TLSO group (*p* = 0.030) (Table 2). No patients in the RCO group progressed to surgery, compared with 32 patients in the TLSO group (*p* = 0.019). Fifteen percent of patients in the RCO group had a final major curve of 45° or greater or progressed to spinal surgery, compared with 38% of patients in the TLSO group (*p* = 0.133). At follow-up, major curves improved by 6° or more in 31% of the RCO group and 13% of the TLSO group (*p* = 0.100).

## Discussion

In this retrospective review of a large academic medical center’s patients with AIS and their experience with full-time brace treatment, we found that patients treated with RCOs were substantially less likely to progress to spinal surgery and had smaller mean change and smaller percent increase in major curves from treatment initiation through follow-up than patients treated with a TLSO, despite similar baseline characteristics and brace wear time. The outcomes of curve progression less than 45° or progression to surgery and major curve improvement of at least 6° were not statistically different; however, they appeared to favor RCOs. Although previous studies have shown the benefits of bracing [4, 11–13] and the benefits of the RCO for treatment of AIS [7, 8], none has compared efficacy of the RCO with other orthoses. For this study, we incorporated guidelines from the SRS Bracing Committee and SOSORT to establish our inclusion criteria [10] and tracked patients from early Risser stages until maturity or surgery to understand the effects of brace type, specifically RCO versus TLSO, on outcomes.

We consider our outcomes for brace treatment in relation to previous studies’ findings. In the Bracing in Adolescent Idiopathic Scoliosis Trial study, 72% of those with TLSO bracing had curve progression to less than 50° [4]. Similarly, our study showed that 68% of patients with TLSO bracing had major curve progression to less than 45° [4]. Previous studies, mostly using Milwaukee TLSO braces, have shown a large spectrum of success rates for a range of curve outcomes, likely because of dissimilarity in brace quality, patient characteristics, and decision thresholds for spinal surgery [14–18]. Most of these studies took place before SRS and SOSORT guidelines on reporting; thus, standards of outcome measurement and participant selection varied [10].

Little research has been published on outcomes for RCOs. Zaborowska-Sapeta et al. [8] reported on 79 patients with RCOs in Poland. In their study, 12.9% of patients progressed to a major curve greater than 50° at final follow-up, with a mean major curve increase of 9.2° for the overall study population [8]. Although we used the SRS-recommended outcome of 45°, our results are comparable to those of Zaborowska-Sapeta et al. [8]. However, our population had a lower mean change in major curve from baseline to follow-up with the RCO. Ovadia et al. [7] published the results of 93 patients in Israel treated with RCOs and found that 84% of patients’ curves progressed by less than 5°. Although we studied a smaller number of patients with RCOs than these two international reports, our study is an important addition to the research because it is one of the first to compare outcomes after RCO use versus general Boston-style TLSO bracing.

Several factors could have contributed to the favorable outcomes for RCOs compared with TLSOs in our study. First, the RCO construction with three-dimensional corrective forces may have a better effect on scoliosis curves compared with the TLSO. Second, the lighter weight of the RCO and more open design may have made it more desirable and comfortable for patients to wear, leading to increased compliance. However, we did not observe a difference in patient-reported mean wear time between brace types during the course of follow-up. Third, because this was an observational study of clinical practice, families had a choice of orthotists and orthoses. Although we did not measure how families made these decisions, we believe variation in geographical distance to orthotists and heterogeneity of insurance coverage for orthoses could have influenced the type of brace adolescents received. In addition, families who chose the RCOs could have had other factors that made their adolescents more likely to have successful bracing outcomes.

This study has limitations. Despite the large number of records encompassing 15 years of a busy, academic scoliosis clinical practice, we had a relatively small sample of patients using RCOs compared with the two international reports, and this may have limited our ability to detect statistically significant differences in some measures [7, 8]. RCO braces were principally made by one skilled orthotist in the region, which contributed to their lower frequency. Despite this, the relative comparability of our outcomes with previous TLSO and RCO studies provides face validity. In addition, this was a retrospective review of an outpatient clinical practice, and we did not have quality-of-life measures, objective monitoring of time wearing the brace, or blinded, independent outcome assessment. Although self-reports tend to overestimate brace wear time [19], it is unlikely that reported wear time would differ systematically between patients with TLSOs and RCOs in this review of a real-world clinical practice.

Another potential limitation was that although the percent initial in-brace major curve correction appeared to be better in RCOs compared with TLSOs, the difference was not statistically significant, as we may have expected given the positive RCO outcomes at the end of treatment. This could have been caused in part by the smaller number of RCOs and by the fact that if the initial correction was not clinically acceptable to the orthopedist, he would recommend the patient return for brace adjustments to achieve optimal correction. Further, in-brace radiography was generally not performed. Thus, the 1-month in-brace measurements presented here may underestimate actual in-brace correction, particularly for RCOs. In addition, the in-brace measured curve correction reflects coronal changes only, not rotational changes, which could not be studied. However, on clinical assessment such as out-of-brace examination of forward bending, the orthopedist noticed that rotational prominence often diminished in RCO-treated patients. The RCO’s influence on curve derotation may be particularly important for its effectiveness in treating scoliosis; however, future research is needed to elucidate how this mechanism contributes to bracing success [7].

There are several strengths of our report. We followed guidelines for patient inclusion and choice of clinical outcome variables [9, 10]. Our results provide a real-world comparison of patient experience with brace types in a large outpatient scoliosis practice. This use of SRS and SOSORT criteria to compare outcomes by brace type is rare in prior studies. Moreover, the similar clinical characteristics at baseline allow an assessment of differences between brace types, despite a relatively small sample size for the RCO group.

## Conclusions

In this large retrospective review of an academic outpatient scoliosis practice, patients treated with RCOs were substantially less likely to progress to spinal surgery than those treated with Boston-style TLSOs. Patients treated with RCOs also had smaller mean change and smaller percent increase in major curves from treatment initiation through follow-up. Future studies should examine differences in outcomes by brace type in other settings and in larger samples, and they should investigate the impact of the rotational dimension of correction with RCOs. Clinicians may consider increasing use of RCOs for AIS.

## Abbreviations

AIS: Adolescent idiopathic scoliosis
RCO: Rigo Chêneau orthosis
SOSORT: Society on Scoliosis Orthopaedic and Rehabilitation Treatment
SRS: Scoliosis Research Society
TLSO: Thoracolumbosacral orthosis
